# Supplementary material for: Alternaria alternata JTF001 Metabolites Recruit Beneficial Microorganisms to Reduce the Parasitism of Orobanche aegyptiaca in Tomato
Source: Biology (Basel). 2025 Jan 23;14(2):116. doi: 10.3390/biology14020116 (PMC11851891; doi:10.3390/biology14020116)
Supplement: Supplementary file 1 [file biology-14-00116-s001.zip › Figures S1 and S2.pdf]

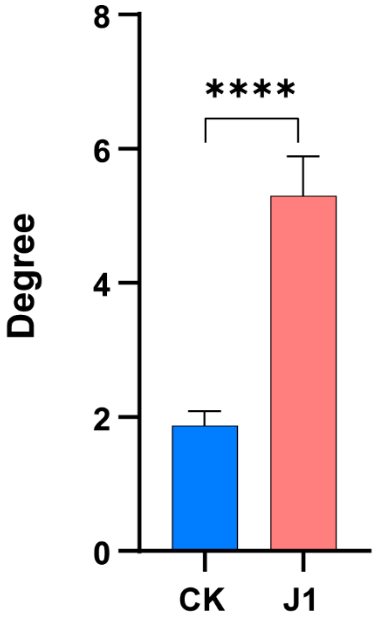

Figure S1. Degree distribution of the inter-kingdom networks. The degree inter-kingdom networks showing the higher complexity of the J1 network than that of the CK network.

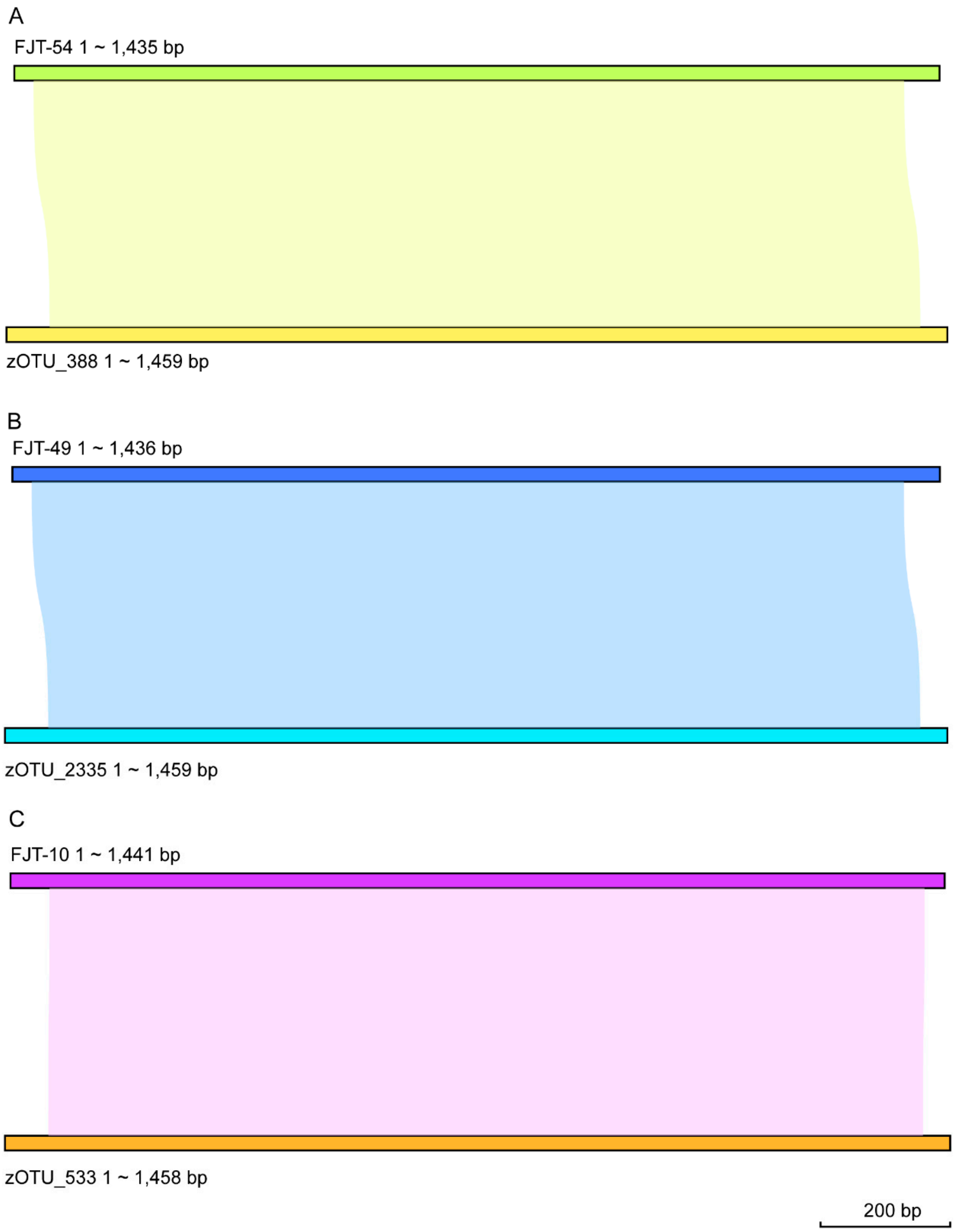

Figure S2. Alignment of the 16S rRNA sequences of the isolate FJT-54, FJT-49, and FJT-10 with zOTU_388, zOTU_2335, and zOTU_533, respectively.
